# A Secondary Metabolism Pathway Involved in the Production of a Putative Toxin Is Expressed at Early Stage of *Monilinia laxa* Infection

**DOI:** 10.3389/fpls.2022.818483

**Published:** 2022-03-24

**Authors:** Maria Villarino, Silvia Rodríguez-Pires, Elena Requena, Paloma Melgarejo, Antonieta De Cal, Eduardo A. Espeso

**Affiliations:** ^1^Grupo Hongos Fitopatógenos, Departamento de Protección Vegetal, Instituto Nacional de Investigación y Tecnología Agraria y Alimentaria (Consejo Superior de Investigaciones Científicas), Madrid, Spain; ^2^Laboratorio de Biología Celular de Aspergillus, Departamento de Biología Celular y Molecular, Centro de Investigaciones Biológicas Margarita Salas-Consejo Superior de Investigaciones Científicas, Madrid, Spain; ^3^Dirección General de Producciones y Mercados Agrarios, Ministerio de Agricultura, Pesca y Alimentación, Madrid, Spain

**Keywords:** brown rot, pathogenesis-related pathways, RNA-seq, necrotroph, pathogen–host interactions, NRPS, epipolythiodioxopiperazine

## Abstract

The necrotrophic pathogenic fungus *Monilinia laxa* causes brown rot disease on stone fruit generating significant yield losses. So far, a limited number of pathogenesis-related virulence factors, such as cell wall degrading enzymes and potential phytotoxins, have been described in *Monilinia* spp. Using RNA-sequencing data from highly virulent *M. laxa* ML8L strain at early stages of the infection process (6, 14, 24, and 48 h post-inoculation, hpi) on nectarine and the Pathogen-Host-Interactions (PHI) database, we selected a number of genes for further study and ranked them according to their transcription levels. We identified a class of genes highly expressed at 6 hpi and that their expression decreased to almost undetectable levels at 14 to 48 hpi. Among these genes we found Monilinia__061040 encoding a non-ribosomal peptide synthase (NRPS). Monilinia__061040 together with other five co-regulated genes, forms a secondary metabolism cluster potentially involved in the production of epipolythiodioxopiperazine (ETP) toxin. Quantitative-PCR data confirmed previous RNA sequencing results from the virulent ML8L strain. Interestingly, in a less virulent *M. laxa* ML5L strain the expression levels of this pathway were reduced compared to the ML8L strain during nectarine infection. *In vitro* experiments showed that liquid medium containing peach extract mimicked the results observed using nectarines. In fact, upregulation of the NRPS coding gene was also observed in minimal medium suggesting the existence of a fruit-independent mechanism of regulation for this putative toxin biosynthetic pathway that is also downregulated in the less virulent strain. These results emphasize the role of this secondary metabolism pathway during the early stage of brown rot disease development and show alternative models to study the induction of virulence genes in this fungus.

## Introduction

*Monilinia laxa* (Aderhold and Ruhland) Honey is an important necrotrophic plant pathogen causing brown rot in stone fruit (Byrde and Willetts, 1977). So far, different compounds involved in the process of fruit necrosis by this pathogen have been identified. There are phytotoxins in *Monilinia* spp. causing necrosis in nectarine tissues (Garcia-Benitez et al., 2019). Also the exoproteomes of the most virulent isolates of *M. laxa* showed the presence of Nep1, the necrosis and ethylene-inducing peptide 1-like protein (Rodríguez-Pires et al., 2020b). Phytotoxins and Nep1 have already described as virulence factors in *Botrytis cinerea* (Collado et al., 2000; Schouten et al., 2008; Dalmais et al., 2011). De Cal et al. (2013) showed the relationship between gluconic acid content and acidification of peaches and nectarines during the infection process of *M. fructicola*, enhancing the expression of pectinolytic enzymes that could facilitate pathogen development on fruit.

Genes for secondary metabolite production, more common in pathogenic fungi than in non-pathogenic fungi, are frequently located in close proximity to each other, forming so-called secondary metabolite clusters (SMCs) (Keller and Hohn, 1997; Brakhage and Schroeckh, 2011; Wiemann and Keller, 2014; Shi-Kunne et al., 2019). Classically, identification of SMCs in the genomes is based on the presence of at least one gene encoding the biosynthetic core, usually either a non-ribosomal peptide synthase (NRPS) or a polyketide synthase (PKS), rather than on their timing of expression which is usually related to the end of exponential growth of the culture (Calvo et al., 2002; Yu and Keller, 2005). Flanking these genes encoding key biosynthetic enzymes are a variable range of genes encoding auxiliary proteins or modifying enzymes, such as transporters, methyltransferases, acetyltransferases, prenyltransferases, oxidoreductases and cytochrome P450 monooxygenases, among other metabolic enzymes. These activities modify the core moiety, normally a peptide or a polyketide, to render one or several final products. In most cases, these SMCs harbor a transcription factor of the zinc-binuclear cluster class that specifically regulates the expression of the cluster (Keller et al., 2005; Sexton and Howlett, 2006). In this work, we have identified an early expressed toxin biosynthetic cluster (EETBC) induced at early times and having a similar structure to that described in other SMCs with an NRPS as the main core. We analyzed differences in EETBC gene expression between *M. laxa* strains that differ in virulence during early infection of stone fruit by transcriptome profiling.

## Materials and Methods

### Fungal Strains and Plant Material

Two *Monilinia laxa* isolates differing in their virulence were used in this study (Rodríguez-Pires et al., 2020b). *M. laxa* single-spore strains 8L (ML8L, with high aggressiveness) and 5L (ML5L, with low aggressiveness) were isolated from a mummified ‘Sungold’ plum fruit from a commercial orchard in Lagunilla (Salamanca, Spain) in 2015. ML8L was deposited in the Spanish Culture Type Collection (CECT 21100), and the genome sequence was recently published (Naranjo-Ortiz et al., 2018). ML8L and ML5L strains were maintained on potato dextrose agar (PDA; Difco, Detroit, MI, United States) at 4°C in darkness for short-term storage and as a conidial suspension in 20% glycerol at −80°C for long-term storage. For conidia production, ML8L and ML5L were grown on potato dextrose agar amended with 20% of tomato pulp (PDA-T, Martini et al., 2016) at 22°C for 7–9 days with a 12 h photoperiod.

Nectarines cv. ‘Venus’ [*P. persica* var. *nucipersica* (Borkh.) Schneider] harvested at harvest (211 Julian days) were used for the pathogenesis and quantitative real-time PCR (qRT-PCR) assays. Fruit were inoculated with conidia of both strains.

### Identification of Pathogenicity-Related Genes in *Monilinia laxa*

Balsells-Llauradó et al. (2020) performed a RNA sequencing analysis during infection of non-wounded mature nectarines by *M. laxa* (ML8L strain). Four different times (6, 14, 24, and 48 h post-inoculation, hpi) were analyzed. RNA sequencing data were deposited in the GenBank Sequence Read Archive under accession number PRJNA610066; GEO: GSE146293. Differential gene expression (DGE) analysis of the *M. laxa* ML8L transcriptome was performed to detect changes throughout the course of infection in mature peach. This analysis served to identify pathogen-specific strategies during the early stages of infection. Genes are considered differentially expressed when *P*-value < 0.05 with a log2-fold-change ≥ 1.

Candidate genes involved in pathogenicity were identified in *M. laxa* ML8L using information from the Pathogen-Host-Interactions (PHI) database (Urban et al., 2017). Putative orthologs of the selected genes were also identified by BlastP searches in other fungal species whose genomes have been sequenced and deposited at the National Center for Biotechnology Information (NCBI). The genomes used were: *M. laxa* ML8L (CECT21100) (Naranjo-Ortiz et al., 2018), *M. laxa* Mlax316 (Landi et al., 2020), *M. fructicola* CPMC (Vilanova et al., 2021), *M. fructigena* gena6 (Bioproject PRJNA707424), *B. cinerea* B05.10 (Van Kan et al., 2017), and *S. sclerotiorum* 1980 UF-70 (Amselem et al., 2011).

To visualize amino acid changes in pathogenicity-related genes between *M. laxa* strains ML8L and ML5L, we used Integrative Genome Viewer (IGV version 2.8.10, Robinson et al., 2011).

### Fruit Inoculation and Experimental Design

Harvested ‘Venus’ nectarines were sterilized as described in Sauer and Burroughs (1986) and placed on racks inside storage boxes which were covered with film. In order to maintain the humidity conditions, the boxes had sterile paper at their base moistened with sterile distilled water (SDW) (no contact with the fruit). Each nectarine was inoculated by applying six drops of 30 μL each of a conidial suspension of ML8L or ML5L strains at 10^6^ conidia mL^–1^ on the surface of non-wounded fruit (Balsells-Llauradó et al., 2020). Fruit were kept in a chamber at 22°C and 100% relative humidity (RH). Two experiments were carried out with four biological replicates of three fruit for each sampling time and strain. Time 0 was considered as the time when the drop containing the conidia dried on contact with the fruit. Subsequent sampling times were at 6, 14, 24, and 48 hpi. As control, inoculations of SDW with 0.1% tween were made on 3 fruit. After each incubation time, peel and pulp tissues (1 cm diameter and deep) encompassing the inoculation sites were collected from 12 individual fruit (four biological replicates of three fruit each) per strain immediately prior to inoculation (time 0) and at 6, 14, 24, and 48 hpi. All samples were immediately frozen with liquid nitrogen before being kept at −80°C until further transcriptional profiling analysis. As control, a sample of the conidial suspension of each strain was collected at the beginning of the experiment.

### Liquid Cultures and Experimental Design

Conidia from *M. laxa* isolates ML8L and ML5L were produced as described above and collected with a solution of 0.01% tween in SDW. Two-ml Eppendorf tubes containing 1 ml of 1% lyophilized peach peel in water (Rodríguez-Pires et al., 2020b) or minimal medium [MM, 1% D-glucose and 5 mM ammonium tartrate, as sources of carbon and nitrogen, respectively (Cove, 1966)] were inoculated with the conidial suspension of each isolate to a final concentration of 10^6^ conidia ml^–1^ and incubated in an orbital shaker in the dark at 120 rpm and 22°C. Mycelial samples were harvested by centrifugation after 3, 6, and 14 hpi.

The peach extract was obtained from the lyophilized peel of peach cv. ‘Placido.’ For preparing lyophilized peach peel, the peel was first separated from the rest of the fruit and lyophilized for 48 to 72 h using a Cryodos-50 lyophilizer (Telstar, Terrassa, Spain). The lyophilized peach was then made into a powder by bead beating using a tissue homogenizer (FastPrep ^®^-24, MP Biomedicals, Madrid, Spain) two times for 30 s at 4 m/s (Rodríguez-Pires et al., 2020b).

### Total RNA Extraction, cDNA Synthesis and Quantitative Real-Time PCR

Two total RNA extraction procedures were used. For mycelia harvested from liquid cultures in MM described above, the protocol based on TRI Reagent ^®^ (Merck KGaA, Darmstadt, Germany) was used. Mycelial samples were frozen in liquid nitrogen at −80°C until RNA extraction. Total RNA from mycelial samples grown in MM was extracted as described in Picazo et al. (2020).

Due to the presence of pigments and carbohydrates in fruit and mycelia samples from peach extract cultures, we used an alternative procedure for total RNA extraction (Rodríguez-Pires et al., 2021). Total RNA was isolated from samples collected at indicated times according to the adapted rapid cetyltrimethylammonium bromide (CTAB)-based protocol. Total RNA was dissolved in RNase-free Milli-Q water, immediately frozen in liquid nitrogen, and maintained at −80°C until further use. RNA concentration and purity were measured using a NanoDrop 2000 spectrophotometer, and RNA integrity was checked by 1.2% agarose gel electrophoresis. Total RNA samples were treated with DNase I (Invitrogen, Fisher Scientific SL, Madrid, Spain) according to the manufacturer’s specifications to remove any remaining genomic DNA. cDNA was synthesized from 1 to 2 μg of total RNA using the SuperScript First-Strand Synthesis System for RT-PCR and oligo(dT) primer (Invitrogen, Fisher Scientific SL, Madrid, Spain).

Real-time PCR was performed as described before in Rodríguez-Pires et al. (2021) using a 7500 Fast Real-Time PCR (Applied Biosystems) using GoTaq qPCR Master Mix (Promega, Promega Biotech Ibérica S.L., Madrid, Spain).

Specific primers were designed for six genes on the *M. laxa* ML8L genome (Naranjo-Ortiz et al., 2018) using Vector NTI (Thermo Fisher, Waltham, MA, United States) (Supplementary Table 1), with a length of 18–23 bp and GC content of 50–60%. We have used previously designed oligonucleotides to amplify the *TEF2* gene from *Prunus persica* (Supplementary Table 1) allowing us to verify the quality of RNA from nectarine-containing samples and the absence of fruit cDNA contamination in non-fruit samples. When possible, at least one intron was included, which allowed the design of the primers at the exon–exon junction, thus minimizing the amplification of contaminant genomic DNA. Amplicon sizes ranged between 160 and 250 bp. Each reaction was carried out in triplicate, with a total volume of 20 μl, containing 10 μl of 2 × GoTaq qPCR Master Mix, 7.8 μl of nuclease-free water, 0.6 μl of each primer (10 μM), and 1 μl of cDNA (100 ng/μl). The cycling program was 2 min at 95°C, followed by 40 cycles of 15 s at 95°C and 1 min at 60°C. After the amplification reaction, a melt curve analysis was performed to check the specificity.

### Secondary Metabolite Cluster Prediction

Secondary metabolite gene clusters in *M. laxa, M. fructicola, M. fructigena, B. cinerea* and *S. sclerotiorum* (see Table 1 for strain designation) were identified using the antiSMASH (Antibiotics and Secondary Metabolite Analysis SHell) software package version 5.0 for fungi with default settings (Blin et al., 2019). In addition, the candidate *M. laxa* ML8L non-ribosomal peptide synthetase (NRPS, Monilinia__061040) was further analyzed for its domain structure with antiSMASH, InterPro Scan, Pfam, and Non-ribosomal peptide synthetase substrate predictor (NRPSsp^1^).

**TABLE 1:** Putative gene clusters coding for secondary metabolites detected by antiSMASH annotation of *Monilinia* spp., *Botrytis cinerea*, and *Sclerotinia sclerotiorum*.

Features*	Number of clusters					
	*M. laxa* ML8L	*M. laxa* Mlax316	*M. fructigena* gena6	*M. fructicola* CPMC6	*B. cinerea* Bc05.10	*S. sclerotiorum* 1980 UF-70
Terpene	4	1	4	4	4	3
NRPS	9	7	8	10	11	7
T1PKS	14	6	10	17	9	15
T3PKS	1	1	1	1	1	1
NRPS-like	4	5	8	7	7	9
Indole		1	1	1	2	1
NAPAA					1	
Other	1	1	1	1	1	
Total protein	33	22	33	41	36	36
Total region_SM	31	21	31	39	35	34

*NRPS, non-ribosomal peptide synthetase; PKS, polyketide synthase; indole alkaloids.

### Data Analysis

Data from ETP-cluster gene expression in fruit were calculated using the 2^–ΔΔCt^ method (Schmittgen and Livak, 2008) and normalized to the expression of the constitutive gene of *M. laxa* histone H3 (BK012065) (Rodríguez-Pires et al., 2021). In all cases, a non-template control (NTC) was included using DNase free water instead of DNA. Data were analyzed by analysis of variance (ANOVA) using the STATGRAPHICS program (XVII Centurion. v. 17.2.00). When the *F* test was significant (*p* ≤ 0.05), a comparison of means was made by Tukey HSD Test.

## Results

### Identification of Early-Modulated Genes During Nectarine Infection by *Monilinia laxa*

**FIGURE 1:**
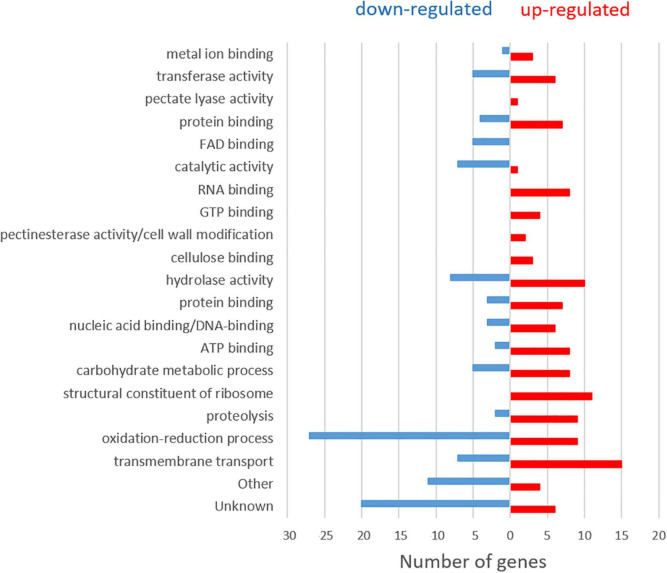
Classification according to gene ontology (GO) terms of the 100 upregulated and 100 downregulated genes with the largest expression difference (*P*-adj ≤ 0.05) between the two sampling times (6 hpi and 14 hpi) in *Monilinia laxa* ML8L.

A general analysis of the expression levels obtained in the RNA-seq of *M. laxa* ML8L during a nectarine infection provided an overview of possible genetic strategies of this pathogen (Balsells-Llauradó et al., 2020). *M. laxa* differential gene expression was detected by comparing the expression profiles of the fungus at each time point against 6 hpi for immature and mature fruit, respectively. In mature fruit, *M. laxa* showed a change in gene expression pattern between 6 and 14 hpi, when disease symptoms were first noticed on the fruit surface (Balsells-Llauradó et al., 2020). Here, these data were analyzed to find possible differences in gene expression during early stages in the infection process. For this purpose, *M. laxa* differentially expressed genes (DEGs) were searched to compare 6 hpi and 14 hpi RNA-sequencing data (PRJNA610066) from Balsells-Llauradó et al. (2020). Of the total 9,567 protein-coding genes predicted in *M. laxa* ML8L (Naranjo-Ortiz et al., 2018), 7,994 DEGs were detected in this two-point comparison (6 hpi vs. 14 hpi). Of these, 3,922 genes were more expressed at 14 hpi than at 6 hpi (up-regulated), and 4,072 genes showed the opposite pattern (down-regulated). Genes in each group were ordered from highest to lowest significant adjusted *P-*value (*P*-adj ≤ 0.05), to visualize the greatest differences in expression levels between 14 and 6 h. Within these two groups (up and down-regulated), approximately 100 genes in each group with the greatest difference in expression between the two sampling points were chosen and functionally annotated using gene ontology (GO) analysis (Ashburner et al., 2000; Mi et al., 2019), and searches for PFAM domains (El-Gebali et al., 2019). In both groups, slightly more than half of the genes were classified as possibly involved in the pathogen–host interactions because homologs were found in the Pathogen-Host Interactions (PHI) database. In addition, a significant number of genes (>15) encoding carbohydrate-active enzymes (CAZymes) were also identified among the selected DEGs (Supplementary Tables 2, 3). Figure 1 summarizes the results obtained in these searches.

At 6 hpi, *M. laxa* mainly induced a high number of DEGs related to oxidative reduction, followed by hydrolase activity and catalytic activity together with transmembrane transport. As the infection process continued (14 hpi), transmembrane transport increased, genes related to the structural constituent of the ribosome appeared, followed by hydrolase activity, oxidation-reduction and proteolysis processes. In addition, the expression of genes related to carbohydrate metabolism, as polygalacturonases (MlPG1, MlPG2) and ATP binding, also increased. An increase of expression levels for genes encoding other activities, such as RNA and GTP binding, together with pectate lyase activity (MlPNL2), pectinesterase activity (MlPME2, MlPME3)/cell wall modification and cellulose-binding, were found in 14 hpi samples compared to 6 hpi.

Based on pathogenesis/virulence-related information through the PHI database, the list of 100 up- and 100 down-regulated genes were subsequently reduced to 47 and 44, respectively. Up-regulated genes related to pathogenesis/virulence clustered under oxidoreductase activity, transmembrane transport, ATP-biding, hydrolase, carbohydrate metabolic process, the carbohydrate-active enzyme (CAZyme) genes CAZYs, and nucleic acid binding (see Supplementary Table 3 PHI column). The group of down-regulated genes related to pathogenesis/virulence were mainly clustered in oxidoreductase activity, followed by hydrolase and catalytic activities (see Supplementary Table 2 PHI column). In general, expression levels of these genes drastically decreased at 14 hpi and remained at very low levels within the following hours of incubation. Some of them were directly inactivated (48 hpi), as is the case of Monilinia__061040 gene with catalytic activity, which had high number of reads at 6 hpi and dropped to practically zero at 14 hpi and afterward.

**FIGURE 2:**
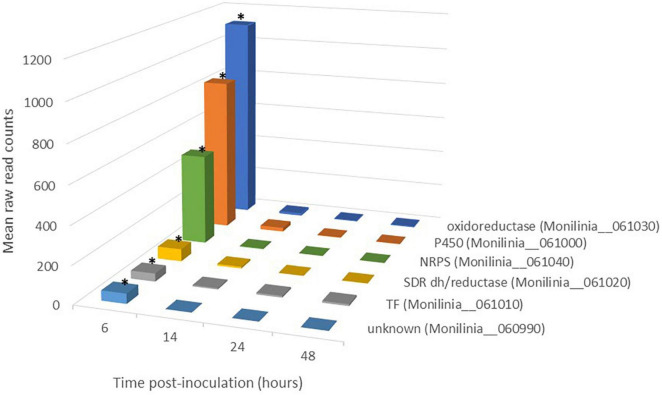
Expression pattern of genes belonging to early expressed toxin biosynthetic cluster (EETBC) in *Monilinia laxa* ML8L during infection of mature nectarine. *Y*-axis indicates the mean raw read counts of *M. laxa* EETBC transcripts in inoculated samples (12 samples) from RNA-seq output in the time point (6, 14, 24, and 48 h). The mean represents the three biological replicates per time point taken for analysis. The bars with an asterisk at 6 hpi are significant with respect to the rest of the times for each gene.

Finally, we selected those genes belonging to pathogenicity-related metabolic pathways, thus reducing the previous list to 31 genes grouped into five pathways (Figure 2, Supplementary Table 4, and Supplementary Figure 1). Among these pathways are those involved in the biosynthesis of oxalic and gluconic acids and the production of reactive oxygen species (ROS). These compounds modify the pH or ROS host response and thus act as virulence factors. Among the genes in this final list are those belonging to two secondary metabolite pathways, one involved in the biosynthesis of the phytotoxin botcinic acid and a second possibly generating a product closely related to the toxin epipolythiodioxopiperazine (ETP). This last pathway has been named as the early expressed toxin biosynthetic cluster (EETBC, see below).

### The *Monilinia laxa* Genome Contains 31 Putative Secondary Metabolite Clusters

Using the antiSMASH software, a prediction of gene clusters in the *M. laxa* ML8L genome was obtained (Table 1). We extended the antiSMASH analysis to the following *Monilinia* genomes (*M. laxa* MLax316, *M. fructigena* gena6 and *M. fructicola* CPMC6) and nearby genera (*Botrytis cinerea* Bc05.10 and *Sclerotinia sclerotiorum* 1980 UF-70) (Table 1). All species have approximately the same number of clusters (31) with *M. fructicola* having the highest number, 39 clusters. Genes encoding biosynthetic backbone enzymes in the clusters in *M. laxa* ML8L included 4 terpene, 9 non-ribosomal peptide-synthetase (NRPS) enzymes, 15 polyketide synthases (PKSs), 2 hybrid PKSNRPSs, and 4 NRPS-like proteins. Similar clusters were found for the rest of the species analyzed (Table 1). In three *Monilinia* species (ML8L, gena6, and CPMC6), the antiSMASH software identified mainly polyketides such as neosartin, neurosporin A, 1,3,6,8-tetrahydroxynaphthalene and botcinic acid as the most similar clusters known, with a similarity of more than 60%. For *M. laxa* Mlax316, 21 clusters were predicted identifying products such as aculeacin A, alternariol, and cichorine with 100% similarity.

The expression levels of all potential NRPS-clusters in *M. laxa* ML8L were analyzed in the RNA-seq data at the different hpi analyzed. The early expression profile (6 hpi) and subsequent down-regulation was not found in any of them (data not shown), except for the predicted NRPS belonging to the identified EETBC pathway.

### Description of the Early Expressed Toxin Biosynthetic Cluster

The EETBC is defined by the Monilinia__061040 gene encoding an NRPS and showing a strong reduction of expression between 6 and 14 hpi on nectarine. The adjacent genes Monilinia__060990 to Monilinia__061030 (Table 2) also showed a similar pattern of expression (Figure 2). Monilinia__060990 has not predicted function, but the others could express accessory proteins involved in the modification of the primary structure of the product synthetized by the NRPS enzyme. Notably, Monilinia__061000 (cytochrome P450) and Monilinia__061030 (oxidoreductase) were highly expressed at 6 hpi compared to 14 hpi, suggesting a mode of regulation. A role that can be accomplished by the transcription factor encoded by DNA-binding binuclear zinc cluster [Zn(II)2Cys6] protein (Monilinia__061010).

**TABLE 2:** Potential early expressed toxin biosynthetic cluster (EETBC) of *Monilinia laxa* ML8L strain.

Gene name	Gene_ID^a^	Protein size (amino acid)	GenBank accession	Predicted protein function^b^
Unknown	Monilinia__060990	221	BK059889	FAM70 unknown function
P450	Monilinia__061000	509	BK059890	P450
TF	Monilinia__061010	757	BK059891	GAL4 + fungal_TF_MHR
SDR dh/reductase	Monilinia__061020	221	BK059892	SRD superfamily (short-chain dh(reductase): NAD-biding)
oxidoreductase	Monilinia__061030	425	BK059893	UbiHs superfamily-FAD-dpte-oxidoreductase
NRPS	Monilinia__061040	1592	BK059894	PP-bin + PRK12316

*^a^Gene unique identifier in the 8L M. laxa genome.*

*^b^Putative protein function based on functional annotation.*

**FIGURE 3:**
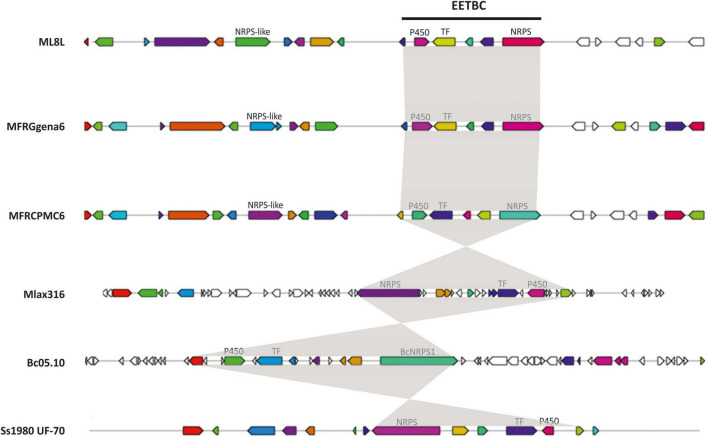
Conservation of early expressed toxin biosynthetic cluster (EETBC) and their genes with functionally characterized homologs. The genes are those characterized in the *Monilinia laxa* (ML8L), *M. fructigena* (gena6), *M. fructicola* (CPMC6), *Botrytis cinerea* (Bc05.10), and *Sclerotinia sclerotiorum* (Ss1980 UF-70) cluster. AntiSMASH used a published database that does not distinguish between full coding sequences and single exons. The shading delimits those genes (indicated as CDS or exons) belonging to the EETB cluster. The colors of the genes are displayed as antiSMASH automatically does. Those genes that are of interest for this work are labeled.

**FIGURE 4:**
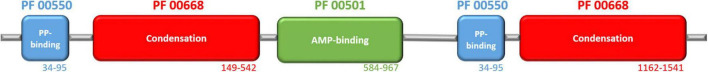
Structure of non-ribosomal peptide-synthetase (NRPS) domains in *Monilinia laxa* ML8L (Monilinia__061040), *Botrytis cinerea* Bc05.10 [BCIN_12g04980 (Bcnrps1)], and *Sclerotinia sclerotiorum* Ss1880 UF-70 (sscle_10g078260). AMP-binding enzyme (PF00501: AMP-binding), condensation domain (PF00668: Condensation), phosphopantetheine attachment site (PF00550: PP-binding) predicted by InterPro Scan.

BlastP searches were conducted using the proteomes of different species of *Monilinia* spp., *B. cinerea* and *S. sclerotiorum* to investigate the conservation of this pathway along species (Table 3). This cluster is not unique to *M. laxa* and showed many similarities and synteny with the other species analyzed. The synteny of the genes within the cluster was conserved in *Monilinia* spp. From the coverage and identity data, it can be seen that it is a highly conserved cluster and that its structure is similar except in *M. laxa* Mlax316 and *S. sclerotiorum* which has a different gene arrangement (Table 3 and Figure 3). The order and orientation of the major genes within the cluster were conserved. Even the order of NRPS, TF and P450 in the closest relative *B. cinerea* was conserved, whereas in *M. laxa* Mlax316 and *S. sclerotiorum*, the order of these genes was not (Figure 3). In this fungal secondary metabolism (SM) cluster, the Monilinia__061040 NRPS is the backbone of the pathway. NRPSs synthesize peptides from core domains or modules. A minimal module consists of three core domains: an adenylation (A), a thiolation (T) or peptidyl carrier protein (PCP) domain which binds the activated substrate to a 4’-phosphopantetheine (PP) cofactor and transfers the substrate to a condensation (C) domain which catalyzes peptide bond formation between adjacent substrates (Finking and Marahiel, 2004). Monilinia__061040 encodes a 1592 amino acids NRPS harboring two phosphopantetheine attachment sites (PF00550: PP-binding), two Condensation domains (PF00668: Condensation), and an AMP-binding enzyme (PF00501: AMP-binding) (Figure 4). NRPS substrate Predictor potentially predicted in the adenylation domain Phenylalanine (F) as substrate. This same domain structure was found in *B. cinerea* [BCIN_12g04980 (Bcnrps1)] and *S. sclerotiorum* [SS1G_08561 (sscle_10g078260)]. BcNRPS1 from *B. cinerea* belongs to the epipolythiodioxopiperazine (ETP) module 2 toxin subfamily (Bushley and Turgeon, 2010), and its homolog was also identified in *S. sclerotiorum* (SS1G_08561) (Graham-Taylor et al., 2020).

**TABLE 3:** Annotation and protein size (amino acid), coverage and identity of potential early expressed toxin biosynthetic cluster (EETBC) genes in different closely related species.

Gene name	*M. laxa* Mlax316^a^	% Coverage	% Identity	*M. fructicola* CPMC6^b^	% Coverage	% Identity	*M. fructigena* gena6^c^	% Coverage	% Identity	*B. cinerea* B05.10^d^	% Coverage	% Identity	*S. sclerotiorum* 1980 UF-70^e^	% Coverage	% Identity
Unknown (Monilinia__060990)	EYC80_001904 221aa	100.00	100.00	MFRU_028g00830 221aa	100.00	94.12	g539.t1	100.00	97.29	BCIN_12g04930 221aa	100.00	82.81	SS1G_08566 (sscle_10g078310) 221aa	100.00	82.35
P450 (Monilinia__061000)	EYC80_001903 509aa	100.00	100.00	MFRU_028g00840 590aa	100.00	97.84	g540.t1	94.00	94.65	BCIN_12g04940 509aa	100.00	92.53	sscle_10g078300 (sscle_10g078300) 508aa	99.00	94.08
TF (Monilinia__061010)	EYC80_001902 839aa	100.00	95.10	MFRU_028g00850 796aa	100.00	91.21	g541.t1	95.00	90.66	BCIN_12g04950 802aa	100.00	80.17	SS1G_08564 (sscle_10g078290) 797aa	100.00	83.69
SDR dh/reductase (Monilinia__061020)	EYC80_001901 221aa	100.00	100.00	MFRU_028g00860 221aa	100.00	96.38	g542.t1	100.00	90.91	BCIN_12g04960 221aa	100.00	87.78	SS1G_08563 (sscle_10g078280) 221aa	100.00	90.05
Oxidoreductase (Monilinia__061030)	EYC80_001900 425aa	100.00	100.00	MFRU_028g00870 425aa	100.00	98.59	g543.t1	100.00	98.35	BCIN_12g04970 424aa	99.00	92.43	SS1G_08562 (sscle_10g078270) 424aa	100.00	94.35
NRPS (Monilinia__061040)	EYC80_001899 1593aa	100.00	99.87	MFRU_028g00880 1592aa	100.00	95.41	g544.t1	100.00	96.30	BCIN_12g04980 (Bcnrps1) 1592aa	99.00	87.26	SS1G_08561 (sscle_10g078260) 1680aa	99.00	86.38

*^a^Gene unique identifier in Mlax316 Monilinia laxa genome; BLAST identity and BLAST coverage.*

*^b^Gene unique identifier in CPMC6 Monilinia fructicola genome; BLAST identity and BLAST coverage.*

*^c^Gene unique identifier in gena6 Monilinia fructigena genome; BLAST identity and BLAST coverage.*

*^d^Gene unique identifier in Bc05.10 Botrytis cinerea genome; BLAST identity and BLAST coverage.*

*^e^Gene unique identifier in S. sclerotiorum 1980 UF-70 Sclerotinia sclerotiorum genome; BLAST identity and BLAST coverage.*

This NRPS identified as ETP toxin module 2, the prediction of antiSMASH and the identification of a transcription factor among them (Monilinia_061010) identified this group of genes as a secondary pathway but being expressed at early times of colonization of nectarine surface by *M. laxa*. Subsequently, we have named this pathway as the early expressed ETP-toxin like biosynthetic cluster (EETBC).

Adjacent to the EETB cluster, antiSMASH analysis also predicted the presence of a gene encoding an NRPS-like in *M. laxa* ML8L (Monilinia__060940), *M. fructicola* (MFRU_028g00780), and *M. fructigena* (g534.t1) (Figure 3). However, co-regulation of this gene and EETBC was not observed in *M. laxa*, and putative orthologs in *B. cinerea* (BCIN_12g05070) and *S. sclerotiorum* (SS1G_08578) were not located close to the EETBC suggesting that this NRPS-like coding gene is not part of the EETBC.

### A Detailed Transcriptional Analysis of the Early Expressed Toxin Biosynthetic Cluster Pathway

The pathogenicity assay with the two strains differing in virulence level was carried out as indicated in M&M. Visible infection symptoms on the fruit at 48 hpi were observed at the point of inoculation of either ML8L or ML5L. Non-inoculated controls never showed necrotic lesions. To further analyze the induction process of genes belonging to the EETB cluster from the very early stages of virulence experimental design, we added two additional sampling times to those taken in the previous RNA-seq analysis. Sample 0h_conidia was the conidia suspension without contact with the fruit. Sample 0h_fruit corresponds to a sample taken at the time when the droplet dried on the fruit.

**FIGURE 5:**
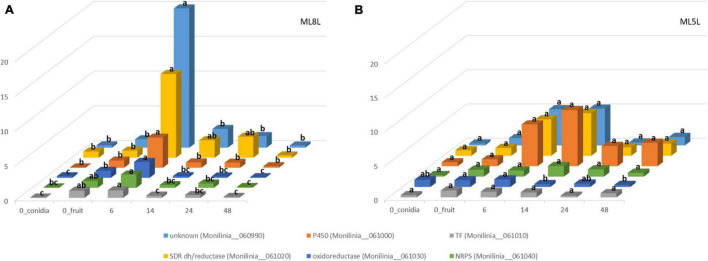
Differential expression of genes coding for early expressed toxin biosynthetic cluster (EETBC). Relative amounts of transcripts for six individual EETBC genes in *Monilinia laxa* ML8L **(A)** and ML5L **(B)**. The amounts of transcripts for each gene at different time points were compared that at 0 hpi_fruit and with histone at the same incubation time [hours post-inoculation (hpi)], using the comparative cycle threshold (*C*_T_) method (2^–ΔΔCt^). Indicated values are the means of triplicates and standard deviations. Each time point represents the mean (*n* = 3) and letters denote significant differences for each gene according to the analysis of variance (ANOVA) and Tukey’s Test. See text for further details about 0h_conidia and 0h_fruit samples.

Differential gene expression data from RNA-seq analysis were validated by performing qRT-PCR on the six genes of the putative EETBC pathway to confirm their expression patterns in pathogenesis assays with *M. laxa* ML8L. Data from qRT-PCR of ML8L (Figure 5A) were consistent with RNA-seq (Figure 2). The expression patterns of the six genes agreed with and validated the results of DGE analysis (Figure 5A). Expression levels at 0 hpi were not high but detectable in the conidia themselves. From 0 hpi to 6 hpi there was a notable increase which indicated that this was an early induction pathway (Figure 5A). The genes in the cluster were coregulated and showed a peak of expression at 6 hpi followed by the rapid decrease at 14 hpi.

Expression of these genes was also studied in the strain ML5L to identify differences in regulation of the EETB cluster between the model virulent strain ML8L and a less virulent strain. In this low virulence strain, increased but not significant expression was also detected at the time of contact with the fruit. Unknown protein (Monilinia__060990), P450 (Monilinia__061000), and oxidoreductase (Monilinia__061030) in ML5L lightly increased their expression at early time points, while NRPS (Monilinia__061040), TF (Monilinia__061010) and reductase (Monilinia__061020) maintained low and constant expression levels throughout the incubation time (Figure 5B). Unlike ML8L, the expression of most of the genes in the ML5L cluster (except for oxidoreductase gene) did not decline after 6 hpi, but maintained the same expression level until 14 hpi (Figure 5B). These data indicated that the ML5L cluster was down-regulated compared to ML8L.

**FIGURE 6:**
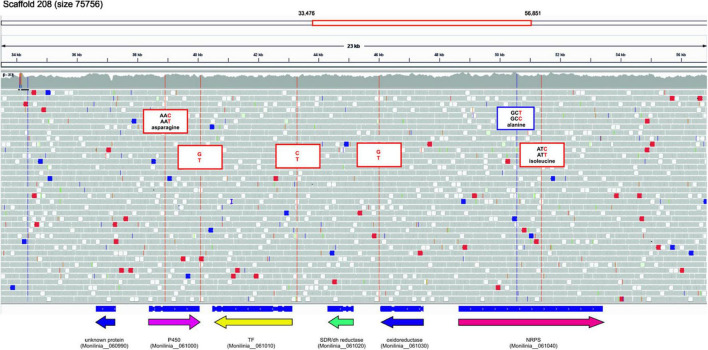
Sequence changes in the early expressed toxin biosynthetic cluster (EETBC) genes of strain *Monilinia laxa* ML5L compared to ML8L (visualized with IGV).

Using the genomic sequence of ML5L strain we found six sequence changes in the EETB cluster (Figure 6). Three single-nucleotide variants (SNVs) were found in coding regions, one in the gene coding Cytochrome P450 and two in the gene coding the NRPS. None of these SNVs caused variations in protein sequence since affected the third base in the codon of respective amino acids (see Figure 6). Two of the SNVs were located in intergenic regions mapped at the terminators of cytochrome P450 and oxidoreductase coding genes (Figure 6). The third non-coding SNV located close to the initiation codon of the transcription factor suggesting a possible effect on its expression levels. However, the qPCR data shown in Figure 5B rule out this possibility, as expression of this gene was recorded at all incubation times. Only minor differences in TF expression levels at 14 hpi were observed between ML8L and ML5L (Figure 5).

### Effect of Peach Extract on the Expression Levels of Monilinia__061040 Non-ribosomal Peptide Synthase

**FIGURE 7:**
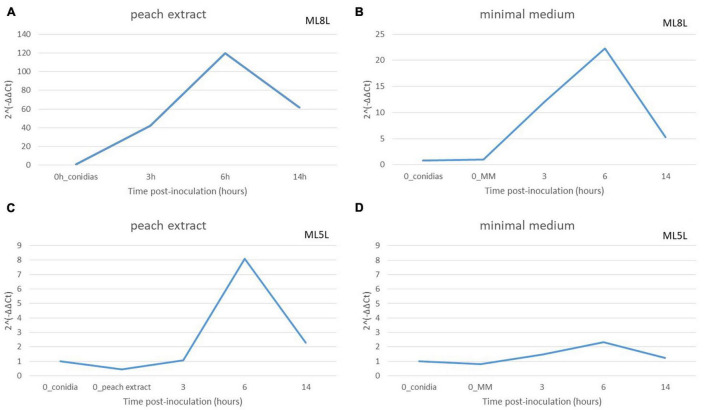
Relative amounts of transcripts for non-ribosomal peptide synthetases enzyme (NRPS) in *Monilinia laxa* ML8L **(A,B)** and ML5L **(C,D)** in different liquid media [peach extract and minimal medium (MM)]. The amounts of transcripts for each gene at different time points were compared that at 0 hpi_conidia and with histone at the same incubation time [hours post-inoculation (hpi)], using the comparative cycle threshold (*C*_T_) method (2^–ΔΔCt^). Indicated values are the means of triplicates and standard deviations. Each time point represents the mean and vertical bars indicate the standard deviation of the mean (*n* = 3) and letters denote significant differences according to the analysis of variance (ANOVA) and Kruskal–Wallis Test.

To define the role of the fruit in the expression levels of the NRPS, these were compared in mycelia grown in minimal medium (MM) versus the same medium containing peach extract. An equivalent amount of ML8L conidia (10^6^ conidia mL^–1^) to that used on nectarine surface was inoculated in liquid MM with or without addition of peach extract. Germination of conidia occurred after 6 h incubation but conidia developed better in MM than in MM added with peach extract. Total RNA was extracted from samples taken after inoculation (0 time) and after 3, 6, and 14 h, and NRPS (Monilinia__061040) expression was evaluated. In both media, Monilinia__061040 was up-regulated at 6 hpi and showed a reduction in expression at 14 hpi (Figures 7A,B). This result suggests that the presence of fruit tissues extracts is not necessary for the activation of NRPS expression, although expression levels increased when the pathogen was in contact with peach extract.

Non-ribosomal peptide synthase expression levels in ML5L were lower than in ML8L but with a similar pattern (Figure 7). The NRPS gene in ML5L showed a peak of expression at 6 hpi in the peach-containing medium, however, this peak was barely detected in MM (Figures 7C,D).

## Discussion

This work identifies a new ETP-toxin like biosynthetic cluster (EETBC) in *Monilinia laxa* early expressed during the infection process. Among the different strategies to understand the infection process, RNA sequencing has proven to be a key tool to understand variations in gene expression patterns during infection (De Miccolis Angelini et al., 2018; Zumaquero et al., 2019; Chittem et al., 2020; Zhang et al., 2020; Ibrahim et al., 2021). Here, RNA-seq results and subsequent quantitative validation through qRT-PCR of six selected DEGs revealed an interesting potential SM biosynthesis cluster in *M. laxa*, which is transcriptionally active during infection of *Prunus persica* cv Venus.

The genome of *M. laxa* ML8L contains 31 putative SM clusters among which five major metabolic pathways were identified as being related to the initiation of the infection process (Supplementary Table 4). Two of them were involved in the biosynthesis of oxalic and gluconic acids, virulence factors whose secretion reduces the pH of host tissues (Maxwell and Bateman, 1968; De Cal et al., 2013). Two other pathways were implicated in the production of virulence toxins, the phytotoxin botcinic acid (Tani et al., 2005, 2006), and the secondary metabolite epipolythiodioxopiperazine (ETP) within the EETBC. Finally, a pathway related to ROS metabolism was identified (Figure 2 and Supplementary Figure 1).

Early expressed toxin biosynthetic cluster organization is based on the Monilinia__061040 gene encoding an NRPS and identified as a *B. cinerea* BcNRPS1 homolog belonging to the epipolythiodioxopiperazine (ETP) module 2 toxin subfamily (Bushley and Turgeon, 2010). That is, EETBC is a secondary pathway but is expressed early in the colonization of the nectarine surface by *M. laxa*, with high levels of expression of its genes at 6 hpi and then switches off at 14 hpi. Notably, this expression pattern contrasts with the expected expression of SM genes during non-exponential growth (Calvo et al., 2002). However, this criteria of late expression of SM clusters is not absolute as it is the case of the pH-dependent regulation of the penicillin cluster in *Aspergillus nidulans* (Espeso et al., 1993). Epipolythiodioxopiperazines (ETPs) are toxic secondary metabolites found only in fungi based on an initial step of condensation of several amino acids, or an amino acid to an acyl-CoA molecule through the activity of an NRPS (Gardiner et al., 2005). In general, an NRPS consists of one or more modules generally composed of three discrete canonical domains (adenylation responsible for amino acid activation (A), thiolation (T) or peptidyl carrier protein (PCP) (also known as thiolation domain). The cofactor 4′-phosphopantetheine (4′PP) binds to the thiolation domain and helps to recruit covalently an activated amino acid, and the condensation domains catalyzes the formation of peptide bonds and releases the peptide (Brakhage, 2013; Soukup et al., 2016). Each module is responsible for the recognition (via the A domain) and incorporation of a single amino acid into the growing peptide product. This NRPS has a domain structure (T-C-A-T-C) similar to that predicted by Interpro for *B. cinerea* and *S. sclerotiorum*. Such similarity predicts that the EETB cluster may well synthetize a similar compound. Searches using Fungi database^2^ showed the presence of possible homologs for Monilinia__061040 in other fungi such as most *Aspergilli*, as previously reported by Graham-Taylor et al. (2020), who also described an NRPS in *S. sclerotiorum*, homologous to BcNRPS1 and the NRPS identified in this work. Sparse information is available about transcriptional regulation of BcNRPS1 homologs, however, in contrast to our findings of early EETBC expression, the *S. sclerotiorum* NRPS was found to be up-regulated at 12, 24, and 48 hpi during *B. napus* infection (Seifbarghi et al., 2017).

The early *M. laxa* ML8L expression profile and its subsequent down-regulation was only found for the predicted NRPS (Monilinia__061040) belonging to the identified EETBC pathway. Timely expression of gene pathways involved in the production of phytotoxins and reactive oxygen species might contribute to the start of infection by necrotrophs (Moraga et al., 2016). The biosynthesis of botrydial and botcinic acid in *B. cinerea* is required for host colonization (Dalmais et al., 2011). The botcinic acid biosynthetic cluster is found in *Monilinia aucupariae*, *M. laxa* and *M. fructicola* (Marcet-Houben et al., 2021). Fruit colonization in the process of *M. fructicola* infection requires local acidification of the host tissue, which is related to the accumulation of gluconic acid (De Cal et al., 2013). Oxalic acid is a pathogenicity factor described in *Sclerotinia sclerotiorum* that plays multiple roles during the infection process (Fagundes-Nacarath et al., 2018; Liang and Rollins, 2018) as for example inducing apoptotic-like programmed cell death in plant hosts (Williams et al., 2011). In these early stages of infection, genes already identified in proteomic studies of the *Monilinia* secretome were also detected (Rodríguez-Pires et al., 2020a,b), such as CAZymes (MlPG1, MlPG2, MlPME2, MlPME3, and MlPNL2) which probably constitute the main enzymatic machinery used by *Monilinia* spp., as well as by *B. cinerea* to penetrate and invade plant tissues (Blanco-Ulate et al., 2014).

The EETBC identified in this work has a similar composition described in other species (Patron et al., 2007) with major elements such as an NRPS (Monilinia__061040), a cytochrome P450 monooxygenase (Monilinia__061000) and a DNA binding binuclear Zn(II)2Cys6-type transcription factor (TF) (Monilinia__061010). This last element is the route-specific regulator. Many gene clusters have a gene for a cluster-specific DNA binding binuclear Zn(II)2Cys6-type TF, which is known to be unique to fungi and activates the transcription of the clustered genes to produce a secondary metabolite (Keller et al., 2005). We identified this TF (Monilinia__061010), as responsible of regulating the production of a potential toxin. This gene showed low levels of expression throughout the assay, although with a significant increase in expression at the first contact with the fruit and at 6 hpi. In filamentous fungi, the activation of specific TFs and the resulting production of secondary metabolites are also controlled by high hierarchy TFs and largely influenced by light (El Hajj Assaf et al., 2020). The VELVET complex comprising the methyltransferase LaeA (loss of *aflR* expression A), VeA (velvet A) and VelB (velvet-like B) proteins, is a global regulator of development and SM in response to light (Bayram et al., 2008). Several authors have found that VeA and LaeA are important regulators of SM-related gene expression (phytotoxin biosynthesis and secreted enzymes) in *B. cinerea* and are therefore required for necrotrophic development. The deletion of BcLAE1 in *B. cinerea* affected the expression of BcNRPS1 and other components of the cluster (Table 3) (Bcin12g04970, Bcin12g04960, Bcin12g04940) (Brakhage, 2013; Schumacher et al., 2015; Müller et al., 2018). LaeA (Monilinia_034020) may be involved in the regulation of the EETBC.

The process of interaction between pathogenic fungi and fruit hosts is complex and generally involves diverse pathogenic-related genes and mechanisms. Different phases of fungal infection on fruit may be considered, but at initial stages secretion of necrosis-inducing proteins to induce local necrosis of host cells is key for successful colonization. Among this machinery are the large range of secreted cell wall degrading enzymes and secondary metabolites to promote cell death (Zhang et al., 2021). We hypothesize that the early expression of the EETB cluster must synthetize a metabolite with a toxin activity, playing an important role in the pathogenesis process. Although the variety of NRPS encoded by fungal genomes is predicted with different computer tools, most of the NRPS-derived metabolites are unknown (Soukup et al., 2016). Through genetic manipulation, some of them have been identified such as gliotoxin, an ETP metabolite that enables the virulence of *A. fumigatus* (Fox and Howlett, 2008), tenellin (Eley et al., 2007), and ochratoxin (Gallo et al., 2012). Recently, the work of Marcet-Houben et al. (2021) identified a number of SM clusters in *Monilinia* species such as those producing tenellin, pyriculol, sordarial, and also monodictyphenone, a prenyl xanthone derivative used as an antifungal and antibacterial compound.

The expression of the EETBC identified in this work does not need contact with the host (fruit) to be expressed. It has been shown that the cluster is also expressed in a more restrictive culture medium such as MM, although the levels are lower. A predetermined programming of expression of this cluster seems to be independent of the host, although the direct contact with the fruit or fruit extract is required for optimal expression of EETBC. Understanding the nature and number of host-dependent pathways that exist in a fungal pathogen is important because they define the range of action of these pathogens. Host-independent pathways can be useful in understanding the general mechanisms of pathogens, with the advantage that they can be studied in more systematically defined settings, regardless of the temporality of the host.

The results obtained from the differences in expression between the two strains exhibiting different degrees of virulence showed the down-regulation of this pathway in the less virulent ML5L strain. The ML5L strain shows an altered expression pattern of this cluster compared to ML8L. The altered expression of genes belonging to the hexadehydroastechrome (HAS)-NRPS gene cluster in *A. fumigatus* showed the biosynthesis of alternative products (Yin et al., 2013). Similarly, in ML5L strain these differences in EETBC expression pattern might cause differences in the quantity or final product composition. Future functional and biochemical studies will elucidate the final products made by EETBC. The characteristics of EETBC expression in strain ML5L support the hypothesis of Rodríguez-Pires et al. (2020b) that brown rot infection and colonization process by weak isolate ML5L might be delayed. This strain showed peach attack initiation problems (germination, infection, and production of extracellular proteins) that could underlie its low virulence compared to other strains (Rodríguez-Pires et al., 2020b). The expression levels of this fungal pathway in contact with the fruit are low and in liquid culture medium are even lower than in ML8L. The ML5L mutation in the TF promoter region can interfere with developmental regulation, stress response, toxin synthesis and pathogenicity as demonstrated in TF mutants of *F. graminearum* (Son et al., 2011). Transcriptional factors can interact specifically with cis-acting elements in gene promoter region, and regulate the spatiotemporal expression of target gene (Zhang et al., 2021).

This work shows that at very early stages of infection a number of pathways are activated, some of them, especially the SM-related pathway involved in the production of a putative toxin. Since these extracellular proteins or metabolites are an important part of the machinery of the interactions between necrotrophic pathogenic fungi and fruit hosts in postharvest diseases (Zhang et al., 2021), future studies will focus on the characterization of this and other toxins produced early by *M. laxa* and most likely, due to genomic conservation, by other *Monilinia* species. This line of research would shed light on the pathogenesis process of this pathogen genus. Simultaneous studies with strains of lower infective capacity will help to gain knowledge on the mechanisms of interaction of enzymes and toxins in the pathogenesis process.

## Data Availability Statement

The datasets presented in this study can be found in online repositories. Nucleotide sequence data reported are available in the Third Party Annotation Section of the DDBJ/ENA/GenBank databases under the accession numbers TPA: BK059889–BK059894.

## Author Contributions

AD and EE proposed this research line. AD, PM, and EE searched for funding. AD and EE conceived and supervised the experiments, which were carried out by MV. SR-P and ER helped with the bioinformatics data. AD, EE, and MV wrote the original draft of the manuscript. All authors contributed to the improvement of the text and figures.

## Conflict of Interest

The authors declare that the research was conducted in the absence of any commercial or financial relationships that could be construed as a potential conflict of interest.

## Publisher’s Note

All claims expressed in this article are solely those of the authors and do not necessarily represent those of their affiliated organizations, or those of the publisher, the editors and the reviewers. Any product that may be evaluated in this article, or claim that may be made by its manufacturer, is not guaranteed or endorsed by the publisher.

## Footnotes

1
http://www.nrpssp.com/

2
Fungidb.org
