# Supplementary figures and images for: A Secondary Metabolism Pathway Involved in the Production of a Putative Toxin Is Expressed at Early Stage of Monilinia laxa Infection
Source: Front Plant Sci. 2022 Mar 24;13:818483. doi: 10.3389/fpls.2022.818483 (PMC8988988; doi:10.3389/fpls.2022.818483)

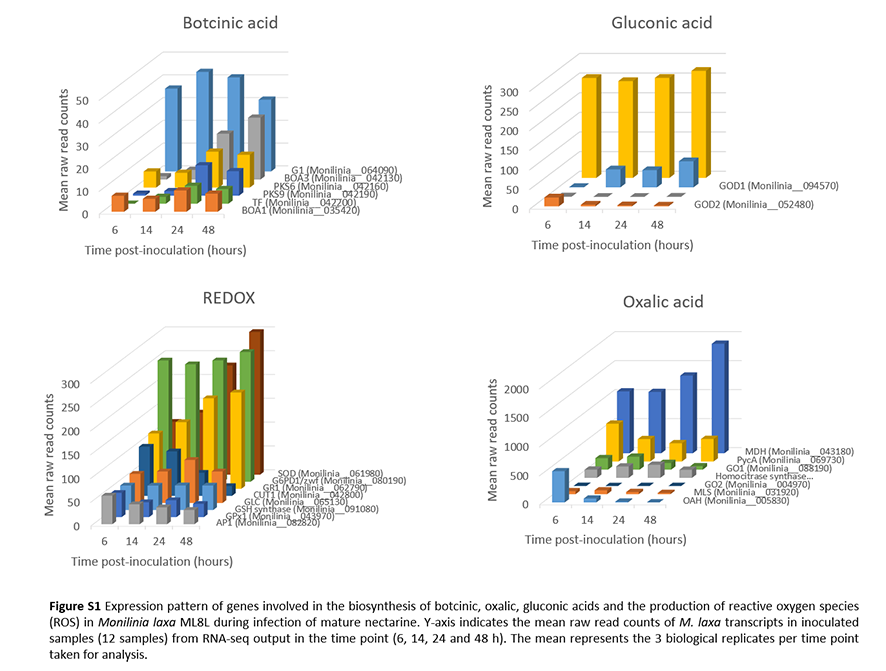

Supplement: Supplementary file 1 [file Image_1.tif]
